# A framework for subjective motion production: A construal approach with Mandarin evidence

**DOI:** 10.1371/journal.pone.0343884

**Published:** 2026-03-02

**Authors:** Xinxin Shan, Zaibing Luo, Gengsheng Xiao, Bin Liu

**Affiliations:** 1 Foreign Languages and Literature, University of South China, Hengyang, Hunan, China; 2 School of Foreign Languages, Southwest Jiaotong University, Chengdu, Sichuan, China; Public Library of Science, UNITED KINGDOM OF GREAT BRITAIN AND NORTHERN IRELAND

## Abstract

This study develops and validates an experimental framework for investigating the production of subjective motion sentences (e.g., *The road winds through the valley*), a phenomenon that has been examined mainly from comprehension perspectives. Using Mandarin as a case study, we first conducted corpus-based analyses to develop the framework, which incorporates: (1) an analysis of four key construal operations (selection, perspective, prominence, and imagination) together with aspectual construals, (2) a classification for subjective motion sentence types based on aspectual marking and subject traversability, and (3) three experiential motivations (perceiving, scanning, and imaging) used to characterize recurrent construal tendencies in production. Guided by the framework, we designed picture-elicitation tasks and conducted production experiments with native Mandarin speakers to test its validity. The findings demonstrate that the framework effectively elicits diverse subjective motion types, capturing language-specific regularities while pointing to cross-linguistically applicable principles of motion construal. By linking corpus evidence to experimental validation, the study provides a systematic and replicable approach that can be extended to future cross-linguistic research on motion and language.

## Introduction

Subjective motion, an intriguing linguistic phenomenon, refers to the conceptualization of static spatial configurations through dynamic motion expressions, as exemplified by Mandarin sentences like *这条路穿过整座城市* (“*The road runs through the whole city*”). These sentences appear in many spoken languages, including English, Japanese, Thai, Spanish, Hindi, Finnish, French and Serbian, and others [1–7]. Such sentences describe scenes where no physical entity actually undergoes a change of location. This strategy involves mental simulation in which speakers project motion onto static scenes during production, while listeners reconstruct this simulated motion during comprehension. In the literature, while Talmy categorizes such phenomena under the broad term “fictive motion” [7,8], we adopt the term “subjective motion” (following Langacker [9] and Matsumoto [2]) to specifically emphasize the conceptualizer’s dynamic mental simulation of static scenes. This perspective is crucial for understanding the aspectual behavior discussed in this study.

Existing research has extensively examined the comprehension of subjective motion from both theoretical [2,7,10] and experimental [11–13] perspectives. While studies of literal motion routinely employ controlled elicitation methods (e.g., Slobin’s Frog Story paradigm; cf. Berman & Slobin [14]), research on subjective motion remains constrained by a fundamental data imbalance: the relative scarcity of naturally produced expressions compared to literal motion counterparts manifests in three methodological deficiencies. First, the heavy reliance on researcher-compiled sentences, coupled with the fact that sparse corpus studies have been predominantly restricted to English [15,16], limits the naturalness of the data and its potential for cross-linguistic generalization. Second, inconsistent data collection procedures across studies severely limit the comparability of findings. Third, the absence of standardized production paradigms hinders the systematic examination of the proposed experiential motivations.

The motion encoded in such expressions is conceptually simulated rather than physically real. Successful elicitation of these sentences requires participants to “perform a particular ‘visual scanning’ of concrete elements of a scene” [4], raising a crucial methodological question: How can experimental stimuli be designed to optimally trigger this mental scanning process and consequently elicit authentic subjective motion expressions? This production-oriented perspective remains substantially underexplored in previous research.

Unlike literal motion research that benefits from established cross-linguistic paradigms, subjective motion studies lack standardized elicitation tools. Although some attempts have been made [4,17], these approaches have proven difficult to replicate across languages and often yield insufficient motion expressions [18]. This methodological gap necessitates the development of adapted elicitation procedures.

This study establishes a framework for investigating how speakers generate subjective motion expressions. Using Mandarin as a case study, we first analyze corpus data to examine how construal operations and aspectual construals shape subjective motion expressions, classify sentence types, and identify correlations between sentence types and potential experiential motivations. The linguistic evidence underpinning our framework comprises three sources: (1) Mandarin equivalents of canonical examples from the literature; (2) elicited materials from preliminary trials; and (3) naturalistic usage examples extracted from the BCC and CCL corpora through targeted keyword searches of linear entities and motion verbs. This triangulation of data sources ensures that the proposed framework captures both theoretical distinctions and naturalistic usage patterns. Finally, we validate the framework through controlled production experiments, testing its ability to elicit and classify the full range of subjective motion sentences while revealing their cognitive foundations.

## Cognitive construal

*Construal* refers to the relationship between a language user’s conceptualization and their linguistic portrayal of situations [19]. In analyzing subjective motion sentences, we adopt a dual construal approach focusing on: (1) the construal operations activated through linguistic representation, and (2) the aspectual/tense patterns employed in grammatical marking.

### Construal operations

Cognitive linguists have proposed various classifications of construal operations [20–23]. Following Langacker [24], this study examines four key construal operations, namely *selection*, *perspective*, *prominence*, and *imagination*, in analyzing subjective motion sentences. This focused approach enables systematic investigation of how specific operations influence construal choices.

#### Selection.

Selection refers to the cognitive process of attending to relevant aspects of experience while disregarding irrelevant ones. In most cases, different words within a semantic frame direct attention to distinct elements. A crucial aspect of selection is *specificity* (or conversely, *schematicity*), defined as “the level of precision and detail at which a situation is characterized” [21]. In subjective motion, this manifests in varying interpretations of the moving entity (cf. Matsumoto [2]). For example:

(1)

a *这条路穿过隧道。*

**Table:** 

Zhè tiáo	lù	chuān-guò	suìdào.
this CL	road	pass through	tunnel
“The road passes through a tunnel.”

    b *(我开的）这条路刚穿过了隧道。*

**Table:** 

(Wǒ kāi de)	zhè tiáo	lù	gāng	chuān-guò-le	suìdào.
I drive DE	this CL	road	just then	pass through-PFV	tunnel
“The road (I was driving on) passed through a tunnel just then.”					

Sentence (1a) describes motion involving either an arbitrary mover or simply the focus of attention, without reference to any particular time. In contrast, sentence (1b) implies a specific mover (the speaker) experiencing a specific motion at a specific time.

This principle further extends to alternative descriptions of the same situation. Compare the general motion verb *zǒu* (“go”) in (2a), which presents a schematic representation of motion, with the more expressive verb *pánxuán* (“wind”) in (2b), which precisely depicts the path’s sinuous shape:

(2)

a *这条小路沿着山腰走。*

**Table:** 

Zhè tiáo	xiǎolù	yánzhe	shānyāo	zǒu.
this CL	path	along	mountainside	go
“This path goes along the mountain.”

    b *这条路沿着山腰盘旋。*

**Table:** 

Zhè tiáo	lù	yánzhe	shānyāo	pánxuán.
this CL	road	along	mountainside	wind
“This path winds along the mountain.”				

Another attentional contrast in construal involves the mode of scene *scanning*, which refers to the cognitive process by which visual or other perceptual information is integrated to form conceptual representations. Langacker [10] distinguishes between summary scanning and sequential scanning as different cognitive processes that structure complex scenes in experientially distinct ways. Summary scanning underlies the difference between sentence predication and nonpredicated states of affairs, whereas sequential scanning represents the state/process construal. While these scanning modes differ from subjective motion, they nevertheless offer potential explanations for subjective motion phenomena. Consider the following examples:

(3)

a *一条羊肠小道从山脚延伸到山顶。*

**Table:** 

Yī tiáo	yángchángxiǎodào	cóng	shānjiǎo	yánshē-dào	shāndǐng.
one CL	narrow winding trail	from	mountain foot	extend-arrive	mountain top
“A narrow winding trail extends from the mountain foot to the top.”

    b *一条羊肠小道从山顶延伸到山脚。*

**Table:** 

Yī tiáo	yángchángxiǎodào	cóng	shāndǐng	yánshē-dào	shānjiǎo.
one CL	narrow winding trail	from	mountain top	extend-arrive	mountain foot
“A narrow winding trail extends from the mountain top to the foot.”

    c ? *一条羊肠小道延伸到山顶，从山脚开始。*

**Table:** 

Yī tiáo	yángchángxiǎodào	yánshēn-dào	shāndǐng,	cóng	shānjiǎo	kāishǐ.
one CL	narrow winding trail	extend-arrive	mountain top	from	mountain foot	start
“A narrow winding trail extends to the top from the foot of the mountain.”						

The semantic distinction between (3a) and (3b) reflects different directions of summary scanning. The conceptualizer mentally traces the trail’s configuration by scanning along its extension in either an upward (3a) or downward (3b) direction, as specified by the *cóng* (“from-”) and *dào* (“to-”) phrases. The linear order of linguistic elements naturally guides our conceptual processing sequence. We first encounter the starting point (*cóng* phrase) and then the endpoint (*dào* phrase), creating optimal processing alignment between linguistic and conceptual sequencing.

Example (3c) demonstrates a processing conflict where these sequences are misaligned. The scanning path begins at the mountain foot conceptually, yet the word order initially directs attention to the mountaintop endpoint. As Langacker [21] observes, such non-alignment requires additional cognitive effort, as the conceptualizer must retrospectively reorganize the scanning sequence after initial processing to properly comprehend the spatial configuration.

Sequential scanning, by contrast, involves dynamic cognitive operations where the conceptualizer performs continuous mental transformations, successively building and modifying representations to simulate motion along a path. This process enables the conceptualizer to construct coherent, contiguous representations rather than activating discrete, isolated points or locations. The importance of sequential scanning becomes evident when examining path length effects:

(4)

a ?? *这条道从这儿延伸到那儿。（五米长）*

**Table:** 

Zhè tiáo	dào	cóng	zhè’er	yánshēn-dào	nà’er.	(wǔ mǐ cháng)
this CL	path	from	here	extend-arrive	there	(five meter long)
?? “The path goes from here to there. (five meters long)”

    b *这条道从这儿延伸到那儿。（五百米长）*

**Table:** 

Zhè tiáo	dào	cóng	zhè’er	yánshēn-dào	nà’er.	(wǔbǎi mǐ cháng)
this CL	path	from	here	extend-arrive	there	(five hundred meter long)
“The path goes from here to there. (five-hundred meters long)”						

Sentence (4a) is problematic because the path’s brevity (five meters) offers insufficient scope for meaningful sequential scanning, making dynamic construal difficult. In contrast, (4b) with its substantial path length (five hundred meters) readily supports sequential scanning operations, allowing the conceptualizer to progressively trace the path’s extension over a simulated temporal interval.

#### Perspective.

Perspective is “the way the scene is viewed” [21] and is widely regarded as “the most obvious and most commented upon of the construal operations” [20]. This aspect of construal is fundamentally shaped by the *viewing arrangement*, which refers to the cognitive relationship between the *subject* of conception (S) (the conceptualizer) and the *object* of conception (O) (the entity being conceptualized). This relationship is inherently asymmetrical. While the object occupies the “onstage” region as the focal point of attention, the subject itself remains unattended. The immediate scope (IS), namely the portion of the conceptualized scene most relevant to comprehension, is situated within a broader maximal scope (MS), which encompasses all potential aspects of the scene.

A key component of the viewing arrangement is the *vantage point*, which is typically anchored to the speaker’s actual location by default. However, the same objective situation can be construed from multiple vantage points, leading to distinct linguistic representations with observable consequences. In other words, spatial relations in language are inherently tied to the speaker’s situatedness. Consider the following contrast:

(5)

a *这是一条来学校的路。*

**Table:** 

Zhè shì	yī tiáo	lái	xuéxiào	de	lù.
this be	one CL	come	school	DE	road
“This is a path that comes to the school.”

    b *这是一条去学校的路。*

**Table:** 

Zhè shì	yī tiáo	qù	xuéxiào	de	lù.
this be	one CL	go	school	DE	road
“This is a path that goes to the school.”					

These examples illustrate *deixis*, a phenomenon that leverages the speaker’s spatial situatedness to structure meaning. While deixis has been extensively studied [25,26], our focus here is on its role in construal. The deictic verbs *lái* (“come”) and *qù* (“go”) encode directionality relative to the speaker, productively marking whether the figure is conceptualized as moving toward or away from the speaker.

Alternative construals of static scenes can also be achieved by *scanning*, as seen in (3a) and (3b). Visual scanning, whether through head or eye movement, allows the conceptualizer to traverse a scene sequentially. This process introduces the distinction between *subjective* and *objective* construal (and correspondingly, *subjectivity* and *objectivity*).

In (3a) and (3b), *shānlù* (“the mountain road”) is construed subjectively: despite the absence of motion, the conceptualizer mentally simulates movement along the path, either upward or downward. The semantic contrast thus resides not in the physical scene but in the direction of mental scanning, which the viewer imposes to construct a dynamic representation of an otherwise static configuration.

#### Prominence.

The third construal operation is prominence or salience in Langacker’s terminology [21], an abstract yet crucial parameter that governs various semantic asymmetries in language. Among the many types of prominence with linguistic significance, two stand out as being especially important for grammatical structure: *figure-ground alignment* and *profiling*.

In spatial relations, whether describing location or motion (both literal and figurative), language encodes the position of a figure (the entity whose location or movement is being described) relative to a ground (the reference point used to situate the figure). Multiple ground objects may sometimes be invoked. Talmy [7] identifies several properties that predispose an entity to be construed as either figure or ground in spatial relations, including size, mobility, and perceptual salience. This principle also applies to subjective motion sentences, as illustrated below:

(6)

a *一根电线沿着围墙走。*

**Table:** 

Yī gēn	diànxiàn	yánzhe	wéiqiáng	zǒu.
one CL	electric wire	along	wall	go
“The electric wire goes along the wall.”

    b ?? *一堵围墙沿着电线走。*

**Table:** 

Yī dǔ	wéiqiáng	yánzhe	diànxiàn	zǒu.
one CL	wall	along	electric wire	go
?? “The wall goes along the electric wire.”				

Compared to *wéiqiáng* (“the wall”), *diànxiàn* (“electric wire”) is smaller, less immediately perceivable, more movable, more dependent, and thus more readily construed as the figure.

In the following pairs, the two nominals exhibit comparable cognitive attributes as inherently linear, stationary entities of similar dimensions, both constituting fundamental spatial representations in human cognition. As a result, either construal is semantically acceptable. However, the choice of figure and ground influences conceptualization:

(7)

a *路沿着铁轨走。*

**Table:** 

Lù	yánzhe	tiěguǐ	zǒu.
road	along	railway track	go
“The road goes along the railway track.”

    b *铁轨沿着路走。*

**Table:** 

Tiěguǐ	yánzhe	lù	zǒu.
railway track	along	road	go
“The railway track goes along the road.”			

In (7a), *tiěguǐ* (“railway track”) acts as the ground, which serves as a reference point with known location for establishing the location of the figure, *lù* (“road”). In contrast, in (7b), the second-appearing nominal *lù* is given ground status with respect to *tiěguǐ* as figure. This demonstrates how prominence assignment shapes linguistic construal even when the scene remains unchanged.

The second type of prominence is *profiling*. As Langacker [21] explains, every expression derives its meaning by selecting a particular body of conceptual content, termed its “conceptual base”. This base can be understood at two levels. In its broad sense, it encompasses the maximal scope, which refers to the full range of conceptual content across all relevant domains. More narrowly, it comprises the immediate scope, which refers to the specific portion of conceptual content that is placed “onstage” as the focus of attention.

Langacker [27] elaborates that within this scope of conceptual content, each expression highlights a particular substructure as its focal point. This foregrounded element, called the profile, serves as the central object of conception that the expression designates, representing the most salient aspect of the conceptualization.

**Fig 1:**
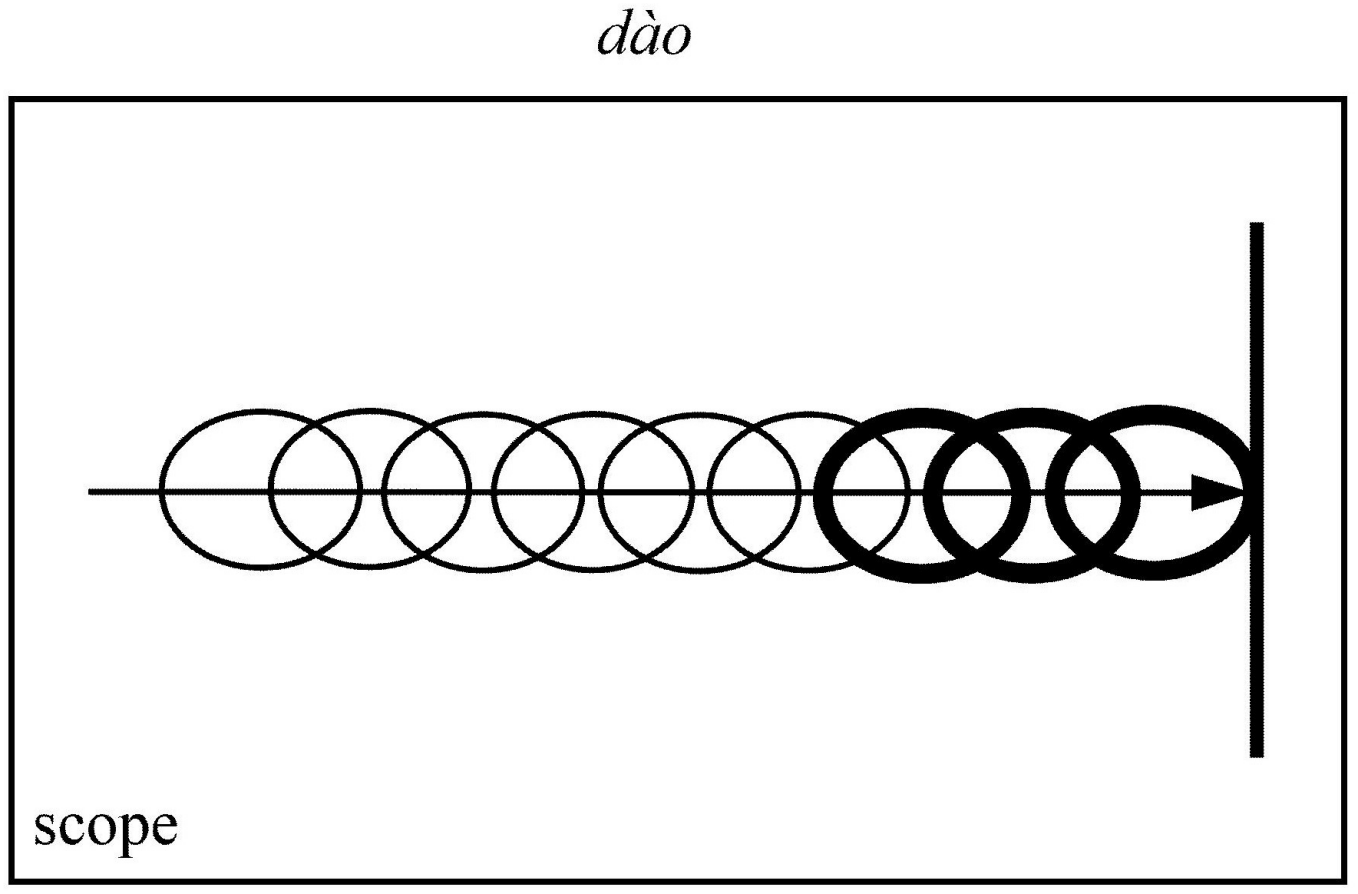
Profiling: Lexical examples (Based on Langacker [24]).

To illustrate how profiling operates in actual language use, we may examine both lexical and grammatical examples. The Mandarin verb *dào* (“arrive”) provides a lexical illustration: while it activates the complete conceptualization of an entity traversing a spatial path toward a destination, it selectively profiles solely the terminal phase of this trajectory (Fig 1).

This fundamental principle of profiling demonstrates how the same objective situation can be conceptualized through multiple construal patterns, thereby eliciting diverse subjective motion expressions. The cognitive flexibility of profiling allows speakers to selectively highlight different aspects of a static scene, generating varied linguistic representations that reflect alternative perspectives on spatial configuration.

(8)

a *输油管道进入涵洞。*

**Table:** 

Shūyóu guǎndào	jìn-rù	hándòng.
oil pipe	enter-enter	culvert
“The oil pipe enters the culvert.”

    b *输油管道穿过涵洞。*

**Table:** 

Shūyóu guǎndào	chuān-guò	hándòng.
oil pipe	pass through	culvert
“The oil pipe passes through the culvert.”

    c *输油管道从涵洞伸出。*

**Table:** 

Shūyóu guǎndào	cóng	hándòng	shēn-chū.
oil pipe	from	culvert	stretch-exit
“The oil pipe stretches out from the culvert.”

    d *输油管道从外伸到涵洞内。*

**Table:** 

Shūyóu guǎndào	cóng	wài	shēn-dào	hándòng nèi.
oil pipe	from	outsides	stretch-arrive	culvert inside
“The oil pipe goes from outside to the culvert.”				

These four sentences depict an identical static configuration. While each verb evokes the conceptualization of an entity moving along a path, they differ in their profiling patterns: (8a) highlights the goal, (8b) the traversal, (8c) the source, and (8d) both the initial and final proportion of the trajectory.

For grammatical illustration, consider motion verbs with an imperfective marker, a category of aspectual markers that we will discuss in more detail in the following section (e.g., *chuān-guò* “pass through” versus *zài chuān-guò* “passing through”).

(9)

a *这条路穿过隧道。*

**Table:** 

Zhè tiáo	lù	chuān-guò	suìdào.
this CL	road	pass through	tunnel
“The road passes through a tunnel.”

    b *这条路正在穿过隧道。*

**Table:** 

Zhè tiáo	lù	zhèng zài	chuān-guò	suìdào.
this CL	road	right IPFV	pass through	tunnel
“The road is passing through a tunnel.”				

**Fig 2:**
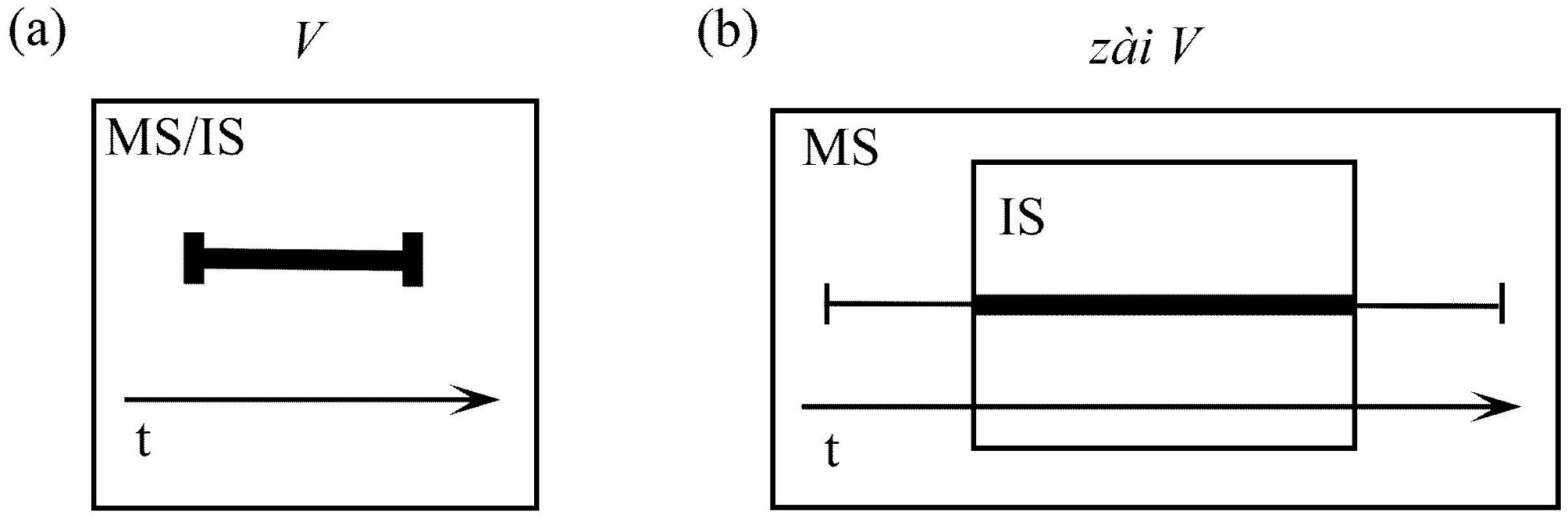
Profiling: Grammatical examples (Based on Langacker [21]).

In (9a), there is no need to distinguish between maximal and immediate scopes, hence the temporal scope is labeled MS/IS. The bold line represents the profiled event, with the entire bounded event, including its initial and terminal points, appearing within the onstage region. By contrast, (9b) exhibits the semantic contribution of the imperfective marker *zài*, which imposes a particular construal on the verbal content. Specifically, it effects a conceptual “zooming in” that establishes a restricted immediate scope excluding the event’s boundaries. The composite expression *zài chuān-guò* (“passing through”) thus maintains a maximal scope encompassing the entire event while limiting the immediate scope to just an internal portion of the temporal progression (see Fig 2).

#### Imagination.

Imagination constitutes a fundamental aspect of construal that permeates numerous aspects of conceptualization. A central component is *mental simulation*, characterized as “the reenactment of perceptual, motor, and introspective states acquired during experience with the world, body, and mind” [28]. When experiencing an event such as entering a tunnel, the brain captures and integrates multimodal states (visual, auditory, kinesthetic, etc.) into a unified representation stored in memory. Subsequent conceptualization reactivates these simulations to reconstruct various aspects of the original experience.

Langacker [24] observes that “even in describing actual situations, the overt object of description is often virtual (or fictive) in nature”. This perspective aligns with empirical evidence suggesting that motion simulation is intrinsically involved in both producing and comprehending subjective motion sentences [11–13,29,30]. Consider the following example:

(10)

*小路穿过隧道。*

**Table:** 

Xiǎolù	chuān-guò	suìdào.
path	pass through	tunnel
“The path goes through the tunnel.”		

In constructing this sentence, speakers mentally simulate either potential moving entities (e.g., people, vehicles) or a focus of attention that conceptually traverses the tunnel’s spatial configuration.

The choice of motion verb constrains the nature of the simulated mover. For instance:

(11)

a *小路驶进隧道。*

**Table:** 

Xiǎolù	shǐ-jìn	suìdào.
path	drive-enter	tunnel
“The path goes into the tunnel.”

       b *小路爬进隧道。*

**Table:** 

Xiǎolù	pá-jìn	suìdào.
path	crawl-enter	tunnel
“The path crawls into the tunnel.”		

In sentence (11a), the verb *shǐ* (“drive/sail”) specifically activates simulations of vehicle movement (cars, buses, ships). Conversely, the verb *pá* (“crawl”) in (11b) evokes simulations of either humans moving on hands and knees (e.g., children) or ground-hugging animals (e.g., worms, caterpillars).

Another widely discussed type of construal is *metaphor*, which also operates within the domain of imagination. The interpretation of subjective motion necessarily incorporates non-linguistic imaginative processes, many of which involve metaphorical mappings. Comprehending manner information in such cases cannot be achieved through conventional semantic analysis alone, but requires explicit recourse to motion-based metaphorical conceptualization. This principle is clearly demonstrated in example (12):

(12)

*铁轨被隧道吞进去了。*

**Table:** 

Tiěguǐ	bèi	suìdào	tūn-jìn-qù le.
railway track	BEI	tunnel	swallow-enter-go LE
“The railway track is swallowed up by the tunnel.”			

In this sentence, the tunnel opening is metaphorically construed as a mouth, while entities entering the tunnel are conceptualized as food being consumed. This metaphorical framework, in which spatial enclosure is conceptualized through the domain of ingestion, enables speakers to produce and understand more creative or unconventional subjective motion expressions that would otherwise defy literal interpretation. The explanatory power of such metaphorical mappings lies in their ability to project structure from concrete, embodied domains (like eating) onto more abstract spatial configurations.

### Aspectual construals

It is important to note that the classification of construal operations presented earlier does not purport to reduce all construals to merely four aspects. In practice, any given sentence involves multiple layers of construal applied to the experience being communicated [20]. Even fundamental conceptual properties, including the categorization of experiences and their basic structure, are subject to construal operations. Consider the following examples:

(13) He **danced** with his girlfriend.

(14) He **was dancing** with his girlfriend when his mother **came in**.

These sentences exemplify two crucial grammatical categories: *aspect* and *tense*. Aspect pertains to different ways of conceptualizing a situation’s temporal structure, while tense concerns the temporal relationship between the situation’s occurrence and the speech moment. In (13), the past tense suffix *-ed* indicates that the dancing event occurred prior to the speech time. The sentences in (14) demonstrate distinct aspectual construals. The second verb complex presents a perfective view of the mother’s entrance, portraying it as a complete, undifferentiated whole without reference to its internal temporal structure. Conversely, the first verb complex adopts an imperfective perspective on the dancing event, focusing on its ongoing duration rather than its beginning or end.

Subjective motion sentences are “inherently imperfective” [30], characterizing static, atemporal situations. Because figures like roads or mountain ranges are expected to remain unchanged indefinitely, overt imperfective aspect marking is generally uncommon in subjective motion expressions. However, several exceptions exist where imperfective aspect becomes appropriate: (1) when depicting figures that are actively extending in real time, (2) when the speaker is simultaneously moving through the physical space being described, and (3) when the speaker wishes to emphasize the transient or temporary quality of the figure [31,32].

Cross-linguistic analysis of construal operations must account for language-specific grammatical resources. While many languages employ both tense and aspect systems to structure temporal conceptualization, Mandarin presents a distinctive case – it lacks grammatical tense markers. This typological characteristic directs our analytical focus toward aspectual construals when examining Mandarin subjective motion expressions. Specifically, we investigate how perfective (*-le*) and imperfective (*zài*/ *-zhe*) markers shape the conceptualization of static spatial configurations as dynamic events.

#### Perfective marker *-le.*

Before proceeding, it is necessary to distinguish the postverbal aspect marker *-le* from the sentence-final particle *le* (written without a hyphen). In this study, *-le* refers exclusively to the postverbal marker. We adopt the view that postverbal *-le* encodes a perfective viewpoint construal rather than past tense or real-world completion [33]. The Mandarin perfective marker *-le* presents a situation from an external viewpoint as a single bounded whole. As Li and Thompson [33] discuss, *-le* is typically licensed when an event is bounded temporally, spatially, or conceptually. They further propose that an event can be construed as bounded in four major ways:

(15)

a By being a quantified event
b By being a definite or specific event
c By being inherently bounded because of the meaning of the verb
d By being the first verb in a sequence

For the purposes of analyzing subjective motion, our discussion focuses specifically on the first two categories.

The first category, a quantified event, is defined as one where the verbal action is delimited by phrases expressing extent, duration, or frequency [33]. In such cases, the perfective marker *-le* is typically obligatory. This is illustrated in example (16):

(16)

*这条电线沿着围墙走了一百米。*

**Table:** 

Zhè tiáo	diànxiàn	yánzhe	wéiqiáng	zǒu-le	yībǎi mǐ.
this CL	electric wire	along	wall	go-PFV	one hundred meter
“The electric wire goes along the wall for one hundred meters.”					

Here, the spatial quantification *yībǎi mǐ* (“one hundred meters”) provides the crucial bounding that licenses *-le* usage, transforming the static spatial configuration into a conceptually bounded unit. This distinction is further illustrated by the contrast between a neutral and a quantified construal in example (17):

(17)

*这条路沿着河边走, 走了大概五百米。*

**Table:** 

Zhè tiáo	lù	yánzhe	hé biān	zǒu,	zǒu-le	dàgài	wǔbǎi mǐ.
this CL	road	along	river bank	go	go-PFV	roughly	five hundred meter
“This road goes along the river for about five hundred meters.”							

In (17), the initial verb *zǒu* (“walk”) simply names the event without marking boundaries, whereas its second occurrence, explicitly bounded by the spatial quantification *wǔbǎi mǐ* (“five hundred meters”), requires the perfective marker *-le*.

The second major category involves definite or specific events. Li & Thompson [33] observe that “an event will also often qualify as bounded if the direct object is understood as a definite noun phrase”. Consider the contrast between (18) and (19):

(18)

*小路爬上了那个山坡。*

**Table:** 

Xiǎolù	pá-shàng-le	nà gè	shānpō.
path	climb-ascend-PFV	that CL	mountain slope
“The path climbs up that mountain.”			

(19)

*小路爬上山坡。*

**Table:** 

Xiǎolù	pá-shàng(-le)	shānpō.
path	climb-ascend(-PFV)	mountain slope
“The path climbs up a mountain.”		

In (18), the demonstrative *nà gè* (“that”) renders the direct object definite, thereby bounding the event and necessitating *-le* to mark specificity. By contrast, (19) omits *-le*, presenting a neutral statement that simply names the event or states a general fact [33]. This distinction is crucial for our analysis: while the presence of *-le* signals a specific, bounded construal, its absence in neutral contexts signals a generic fact-stating construal. Importantly, neither form implies the ongoing, processual characteristic of the imperfective aspect (discussed in the following section). Instead, consistent with Li and Thompson’s observation [33] that judgments on *-le* usage vary based on communicative intent, the distinction reflects the speaker’s subjective construal – choosing to emphasize either a specific bounded occurrence or a general fact.

Furthermore, the absence of *-le* in bounded contexts is often due to functional redundancy. Li and Thompson note that “conditions for the use of perfective *-le* would appear to be satisfied, but yet no *-le* appears”, often because such sentences “contain another element that does the job of ‘perfectivizing’ the verb” [33]. A classic example involves physical motion:

(20)

*他从房子里走到张三那儿。*

**Table:** 

Tā	cóng	fángzi	lǐ	zǒu-dào	Zhāng Sān	nàr.
he	from	house	in	walk-arrive	Zhang San	there
“He walked from his house to Zhang San’s place.”						

In (20), the directional complement (*dào* + Goal) provides strong endpoint information, which renders the postverbal *-le* redundant. This principle extends systematically to subjective motion constructions, as illustrated in Example (21):

(21)

*栅栏一直延伸到山边。*

**Table:** 

Zhàlán	yīzhí	yánshēn-dào	shān biān.
fence	all the way	extend-arrive	mountain side
“The fence goes all the way to the mountain side.”			

Here, the directional phrase *dào shān biān* (“to the mountain side”) explicitly specifies the spatial extent and endpoint of the configuration, thereby providing strong delimiting cues. Consequently, postverbal *-le* can be omitted without loss of the intended endpoint-oriented construal, because the bounding information is already contributed by the directional complement.

#### Imperfective markers *zài* and *-zhe.*

Mandarin employs two distinct grammatical markers to encode imperfective aspect: the preverbal particle *zài* and the suffixal marker *-zhe*. Crucially, the aspectual marker *zài* must be distinguished from its homophonous counterpart in locative phrases. While locative *zài* precedes noun phrases denoting location, aspectual *zài* occurs immediately before activity verbs to indicate ongoing events.

Following Li and Thompson’sclassification, imperfective *zài* is prototypically associated with activity verbs, defined as those denoting “the active participation and involvement of an animate subject in an event” [33]. This verb class includes: prototypical action verbs such as *zǒu* (“walk”) and *dǎ* (“hit”), and non-action activity verbs such as *kàn* (“read or look at”), *xīnshǎng* (“appreciate”), and *xué* (“study”). Conversely, verbs like *shòu* (“lose weight”), *zhīdào* (“know”) and *shōudào* (“receive”) are excluded, as they describe states rather than active participation in some sort of activity.

The verbs employed in subjective motion expressions represent only a subset of Mandarin’s action verbs. These verbs maintain the capacity to combine with the imperfective marker *zài* to denote ongoing events, despite describing conceptually static spatial configurations. Consider example (22):

(22)

*这条路在沿河走。*

**Table:** 

Zhè tiáo	lù	zài	yán	hé	zǒu.
this CL	road	IPFV	along	river	go
“This road is going along the river.”					

In (22), the prepositional phrase *yán hé* (“along the river”) merely specifies the path, while *zài* serves to construe the motion as an ongoing process rather than a completed event.

The suffixal imperfective marker *-zhe* operates differently, primarily functioning to background one durative event against another. As Li and Thompson [33] observe, *-zhe* typically appears “in the first of two clauses to signal that one event provides a durative background for another event”. This pattern is exemplified in the following subjective motion expressions. Based on our preliminary corpus observations, the first verb concatenated with the imperfective marker *-zhe* frequently denotes a durative background for the path’s extension. While a systematic quantification lies beyond the scope of this study, this recurring pattern points to a potential semantic constraint that warrants future investigation.

(23)

a *公路盘旋着通至山顶。*

**Table:** 

Gōnglù	pánxuán-zhe	tōng-zhì	shāndǐng.
driveway	wind-IPFV	go-arrive	mountain top
“The driveway is winding to the summit.”

         b *城墙逶迤着伸向远方。*

**Table:** 

Chéngqiáng	wēiyí-zhe	shēn xiàng	yuǎnfāng.
city wall	wind-IPFV	extend toward	distance
“The city wall is winding toward the distance.”

         c *楼梯旋转着通到楼上。*

**Table:** 

Lóutī	xuánzhuǎn-zhe	tōng-dào	lóushàng.
stairs	wind-IPFV	go-arrive	upper floor
“The stairs are winding to the upper floor.”			

In these examples, *-zhe* creates a dynamic construal where the manner of motion (winding, meandering, spiraling) serves as the durative background for the path’s extension. This construction type is particularly productive in Mandarin for expressing subjective motion with rich manner information, provided the manner description doesn’t itself encode path characteristics.

The systematic use of *zài* and *-zhe* thus provides Mandarin speakers with distinct but complementary strategies for construing ongoing motion events, each contributing differently to the aspectual and perspectival properties of subjective motion descriptions.

#### Perfective and imperfective processes in subjective motion.

The aspectual contrast between perfective and imperfective construals in subjective motion sentences can be explored from another perspective, namely *scope*. Consider the following pairs:

(24)

a *这条公路盘山而上。*

**Table:** 

Zhè tiáo	gōnglù	pán shān ér shàng.
this CL	highway	twist mountain and ascend
“This highway winds around the mountain.”

         b *这条公路（正）在盘山而上。*

**Table:** 

Zhè tiáo	gōnglù	(zhèng) zài	pán shān ér shàng.
this CL	highway	(right) IPFV	twist mountain and ascend
“This highway is winding around the mountain.”			

(25)

a *公路盘旋通至山顶。*

**Table:** 

Gōnglù	pánxuán	tōng-zhì	shān dǐng.
driveway	wind	go-arrive	mountain top
“The driveway winds toward the summit.”

    b *公路盘旋着通到山顶。*

**Table:** 

Gōnglù	pánxuán-zhe	tōng-dào	shān dǐng.
driveway	wind-IPFV	go-arrive	mountain top
“The driveway is winding toward the summit.”			

While each pair describes identical physical configurations, their conceptual representations differ fundamentally due to aspectual marking. The (a) sentences, lacking overt imperfective markers, adopt a maximal scope perspective that encompasses the entire spatial configuration as a timeless, unchanging whole. This global viewpoint allows speakers to conceptualize the complete path-mountain system in its totality, preserving all its sinuous curves and topographic relationships as a single gestalt. Such expressions prove particularly suitable for contexts affording comprehensive visual access, such as map representations or aerial views, where the entire configuration can be apprehended simultaneously.

Conversely, the (b) sentences marked with imperfective aspect (using either *zài* or *-zhe*) reflect a highly restricted scope. This dynamic construal mimics the experience of an observer moving through the landscape, where attention remains focused on currently visible path segments that change from moment to moment. The aspectual marking creates a sense of temporal unfolding, as if the speaker were tracking their progress along the route in real time, with spatial relationships continuously updating throughout the journey.

## Categorization and motivation

### Types of subjective motion sentences

The categorization of subjective motion sentences also serves as a fundamental component in developing our design for motion production. There are two primary classification approaches in the literature.

Matsumoto’s binary classification distinguishes between two semantic types based on motion specificity and aspectual properties [2], which reflect different patterns of *selection* in conceptualization (as discussed in Section 2.1.1). *Type I sentences* employ a steady perspective with global attentional scope, while *Type II sentences* adopt a moving perspective with local scope. Consider the contrast in (26):

(26)

a The highway passes through a tunnel there. (*Type I subjective motion* sentence)
b The highway I was driving on passed through a tunnel then. (*Type II subjective motion* sentence)

Matlock [29] proposes an alternative binary classification based on the characteristics of subject noun phrase. *Type 1 sentences* feature traversable linear entities that metonymically suggest motion, such as *roads* and *paths*, while *Type 2 sentences* contain untraversable extended objects without inherent motion associations, such as *fences* and *walls*. The examples in (27) illustrate this contrast:

(27)

a The trail runs across the valley. (*Type 1 subjective motion* sentence)
b The earthquake fault runs across the valley. (*Type 2 subjective motion* sentence)

Building on these foundations, our study develops a four-way classification system that integrates aspectual properties with subject noun phrase traversability. The first parameter aligns with Langacker’s proposal that subjective motion sentences contain both actual and virtual components, where “the situation in actuality determines the basic aspectual categorization” [10]. In Mandarin, which lacks tense markers, aspectual markers become the primary indicators of these conceptual distinctions. Within this system, the selection of vantage point, particularly the contrast between first-person and third-person perspective, plays a crucial role in shaping aspectual choice and motion construal. The fundamental distinction between perfective and imperfective marking reflects alternative conceptualization strategies, as exemplified in (24). The perfective form in (24a) presents a global, map-like apprehension of spatial configuration through third-person perspective taking, while the imperfective marker in (24b) indicates a local, traveler’s viewpoint that creates an immersive “being there” experience through first-person perspective.

Our classification incorporates traversability as another parameter, though we depart from Matlock’s metonymic account. While traversable entities naturally afford motion simulation, untraversable objects require more abstract construal due to limited sensorimotor engagement. This distinction influences the naturalness of motion interpretations and the availability of perspective options.

Based on these two parameters and our observations from the corpus data, Mandarin subjective motion sentences can be categorized into four types. *Type One* consists of constructions where a traversable entity is marked with imperfective aspect (*zài* or *-zhe*). *Type Two* comprises constructions where a traversable entity is encoded with non-imperfective forms (either the perfective *-le* or unmarked forms). Drawing on the analysis in the previous section, this unified grouping in Type Two is theoretically motivated. Distinct from the imperfective aspect, neither form profiles the internal temporal unfolding of the situation. Instead, as established earlier, the choice between *-le* and unmarked forms is driven by communicative intent **–** the speaker’s subjective preference for specificity versus neutrality [33]. This category includes unmarked forms where bounding is already supplied lexically (e.g., by directional complements such as *dào* + Goal), rendering *-le* redundant. Given that this boundary is fluid and subject to construal, treating them as a single category prevents artificial fragmentation and allows for a robust contrast against the process-oriented imperfective. Similarly, this aspectual pattern also extends to untraversable entities. *Type Three* represents the combination of an untraversable entity with imperfective aspect, whereas *Type Four* features the same entity type but encoded with non-imperfective forms.

These four types are exemplified below:

(28)

a 这条路正在翻越山岭。 (*Type One*: Traversable + Imperfective *zài*)

**Table:** 

Zhè tiáo	lù	zhèng zài	fān-yuè	shānlǐng.
this CL	road	right IPFV	climb-pass	mountain ridge
“The road is climbing over the mountain ridge.”

       b *这条路横穿（了）沙漠。* (*Type Two*: Traversable + Non-imperfective)

**Table:** 

Zhè tiáo	lù	héng-chuān (-le)	shāmò.
this CL	road	horizontal-pierce (-PFV)	desert
“The road traverses the desert.”

      c *电线沿着围墙延伸着。* (*Type Three*: Untraversable + Imperfective *-zhe*)

**Table:** 

Diànxiàn	yánzhe	wéiqiáng	yánshēn-zhe.
electric wire	along	wall	extend-IPFV
“The electric wire is extending along the wall.”

       d *电线绕过（了）围墙。* (*Type Four*: Untraversable + Non-imperfective)

**Table:** 

Diànxiàn	rào-guò (-le)	wéiqiáng.
electric wire	wind-pass (-PFV)	wall
“The electric wire winds around the wall.”		

**Fig 3:**
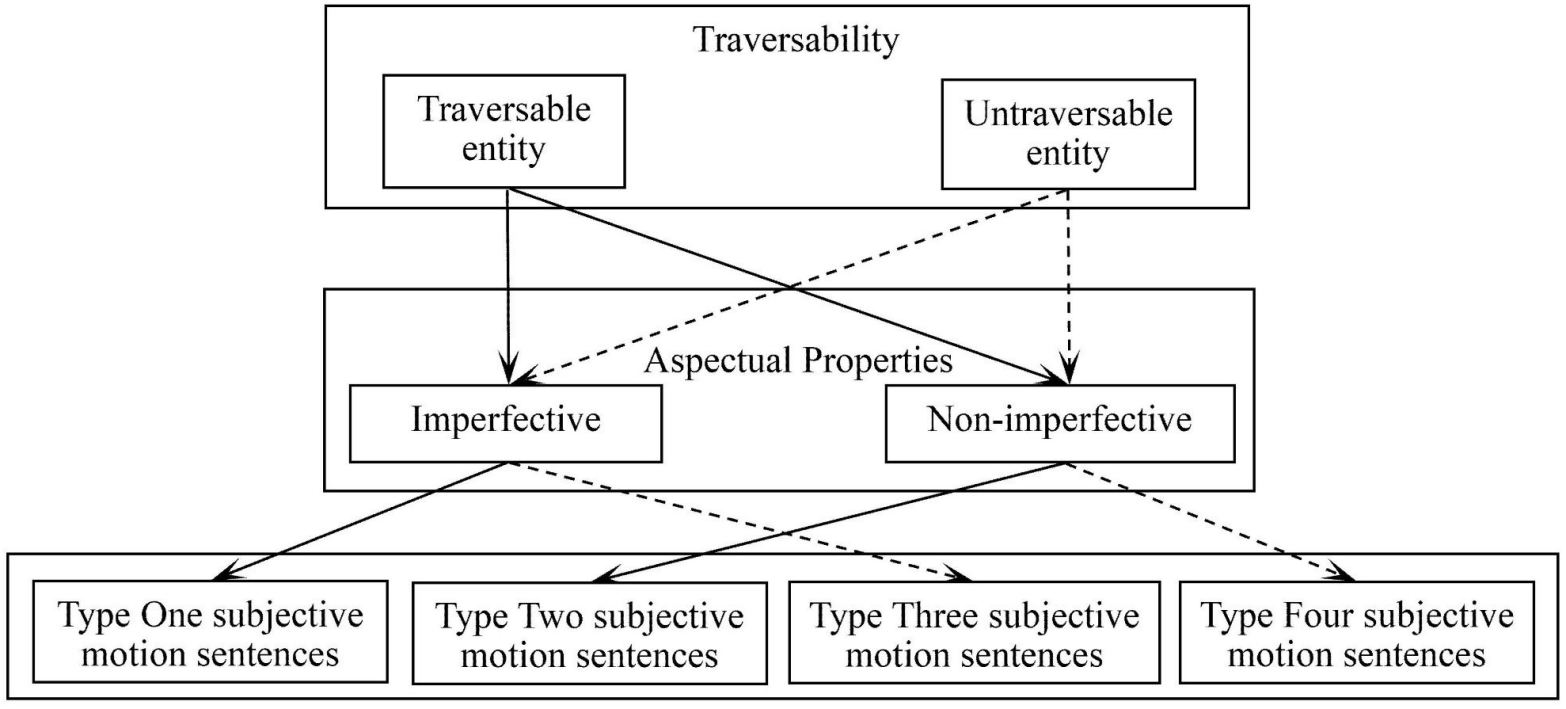
Four types of subjective motion sentences.

The interaction between these two parameters – entity traversability and grammatical aspect – yields four types of subjective motion sentences illustrated in Fig 3.

### Experiential motivations for subjective motion sentences

The phenomenon of subjective motion sentences is fundamentally rooted in diverse motion experiences, reflecting how language structures our conceptualization of spatial relationships. As Croft and Cruse [20] observe, “whenever we utter a sentence, we unconsciously structure every aspect of the experience we intend to convey”. This cognitive principle manifests particularly clearly in subjective motion sentences, where the flexibility in conceptualizing the “moving entity” reflects the diverse experiential bases that motivate these expressions. The linguistic realization of these sentences consistently bears traces of their underlying experiential motivations, though the specific nature of these motivations may vary across different contexts and constructions.

According to Langacker [10], subjective motion sentences like *That mountain range goes from Mexico to Canada* allows multiple interpretations that reflect distinct cognitive processes: the mountain range might be metaphorically construed as moving along a path, or an imaginary mover traversing along the mountain range’s expanse might be posited. His core argument is that “the CONCEPTUALIZER moves SUBJECTIVELY along this path by a process of MENTAL SCANNING”, with these factors operating to varying degrees in different contexts. For example,

(29)

a That mountain range {reaches/ extends/ stretches} from Mexico to Canada.
b This highway goes from Mexico to Canada.
c The freeway ran along the coast for a while, then entered the mountains.
d This road {leads/ takes} you directly to the exit – you just have to follow it.

While in all the sentences the conceptualizer mentally scans the spatial path: a verb choice emphasizes the subject’s subjective motion in (29a), a shifted subject or other adjustments foreground an imagined mover in (29b) and (29c), and (29d) introduces another mover alongside the subject.

This aligns with Talmy’s distinction between constructional fictive (subjective) motion and experienced fictive (subjective) motion [7]. While the former refers to the grammaticalized patterns that encode motion semantics, the latter indicates whether and how individual speakers actually conceptualize motion when processing them. For the same constructional subjective motion, some speakers report strong motion semantics, while others report none. Crucially, every individual experiences motion with *some* subjective motion constructions. Regarding the moving entity, conceptualizations vary across individuals and construction types. Even the same individual may conceptualize the same subjective motion differently on different occasions. For example, when encountering *That mountain range goes from Canada to Mexico*, the conceptualized moving entity varies.

Research on Chinese subjective motion suggests that the phenomenon encompasses two distinct dimensions: (1) expressions which in fact express literal motion, and (2) expressions where motion is construed subjectively via a shifting focus of attention or hypothetical motion [34]. Building on Blomberg and Zlatev’s [17] three experiential motivations for subjective motion sentences – *enactive perception*, *visual scanning* and *imagination of motion* – we adopt the gerund forms *perceiving*, *scanning* and *imaging* for terminological consistency. In the present study, these terms are used as analytic labels for recurring construal tendencies discussed in the literature, not as directly observed online cognitive mechanisms. Accordingly, we treat them as theoretical constructs for organizing predictions about aspectual marking and sentence types, acknowledging that their empirical status remains inferential and requires independent validation with online measures (e.g., eye-tracking).

The motivation of *perceiving* pertains to descriptions compatible with an immersed viewing arrangement, in which the conceptualizer is construed as located within the scene. Such descriptions often foreground an “ongoing” construal and are therefore expected to co-occur with imperfective marking in Mandarin. For example:

(30)

*长长的路正在她眼前展开。*

**Table:** 

Chángchángde	lù	zhèng zài	tā	yanqián	zhankāi.
long	road	right IPFV	her	eye	spread-away
“The long road is spreading out before her eyes.”					

In (30), the road is linguistically construed as a moving entity. This construal aligns with a first-person viewing arrangement in which the conceptualizer is situated as a potential traveler traversing the path.

The motivation of *scanning* pertains to cases in which the description is compatible with an overview construal of an extended configuration. Here, the conceptualizer’s attention is sequentially allocated across the figure’s extent rather than anchored to an immersed journey. This motivation is particularly relevant for descriptions of large-scale or untraversable entities, as well as traversable entities construed from a distanced vantage point. For example:

(31)

*那条山脉从东向西延伸。*

**Table:** 

Nà tiáo	shānmài	cóng	dōng	xiàng	xī	yánshēn.
that CL	mountain range	from	east	toward	west	extend
“That mountain range goes from east to west.”						

(32)

*这条路从山脚盘旋到山顶。*

**Table:** 

Zhè tiáo	lù	cóng	shānjiǎo	pánxuán-dào	shāndǐng.
this CL	trail	from	mountain foot	wind-arrive	mountain top
“The trail winds from the foot to the top of the mountain.”					

Example (31) is interpreted as reflecting attention sweeping across a static landscape, while (32) implies visual tracking from a stationary point, or a map-like view. Both scenarios prioritize a configurational apprehension of the figure’s extent over temporal duration, thereby favoring non-imperfective realizations in Mandarin.

The motivation of *imaging* pertains to cases where speakers elaborate a static configuration by enriching it with imagined motion components. This motivation is proposed to be dominant when entities afford motion and are observed from a third-person perspective. It covers both conventional subjective motion and creative metaphorical extensions. For instance:

(33)

*这条路穿过隧道。*

**Table:** 

Zhè tiáo	lù	chuān-guò	suìdào.
this CL	road	pass through	tunnel
“The road passes through a tunnel.”			

In sentence (33), the expression can be analyzed as inviting a motion simulation that may incorporate a hypothetical mover, the manner of motion, and sometimes terrain information compatible with the scene. Furthermore, as illustrated in example (12) (*“The railway track is swallowed up…”*), metaphorical verb choice can extend motion construal to less canonical configurations, helping to account for some of the more creative or atypical subjective-motion uses.

**Fig 4:**
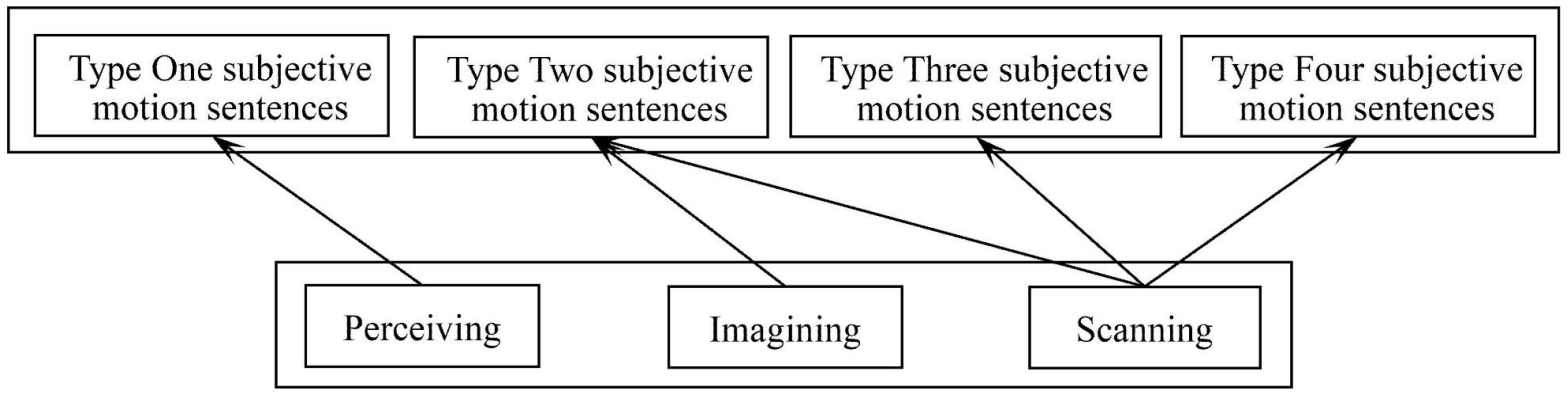
Three experiential motivations for subjective motion sentences.

Fig 4 summarizes the theoretical mapping between these three experiential motivations and the four major categories of subjective motion sentences.

## The production of subjective motion sentences

**Fig 5:**
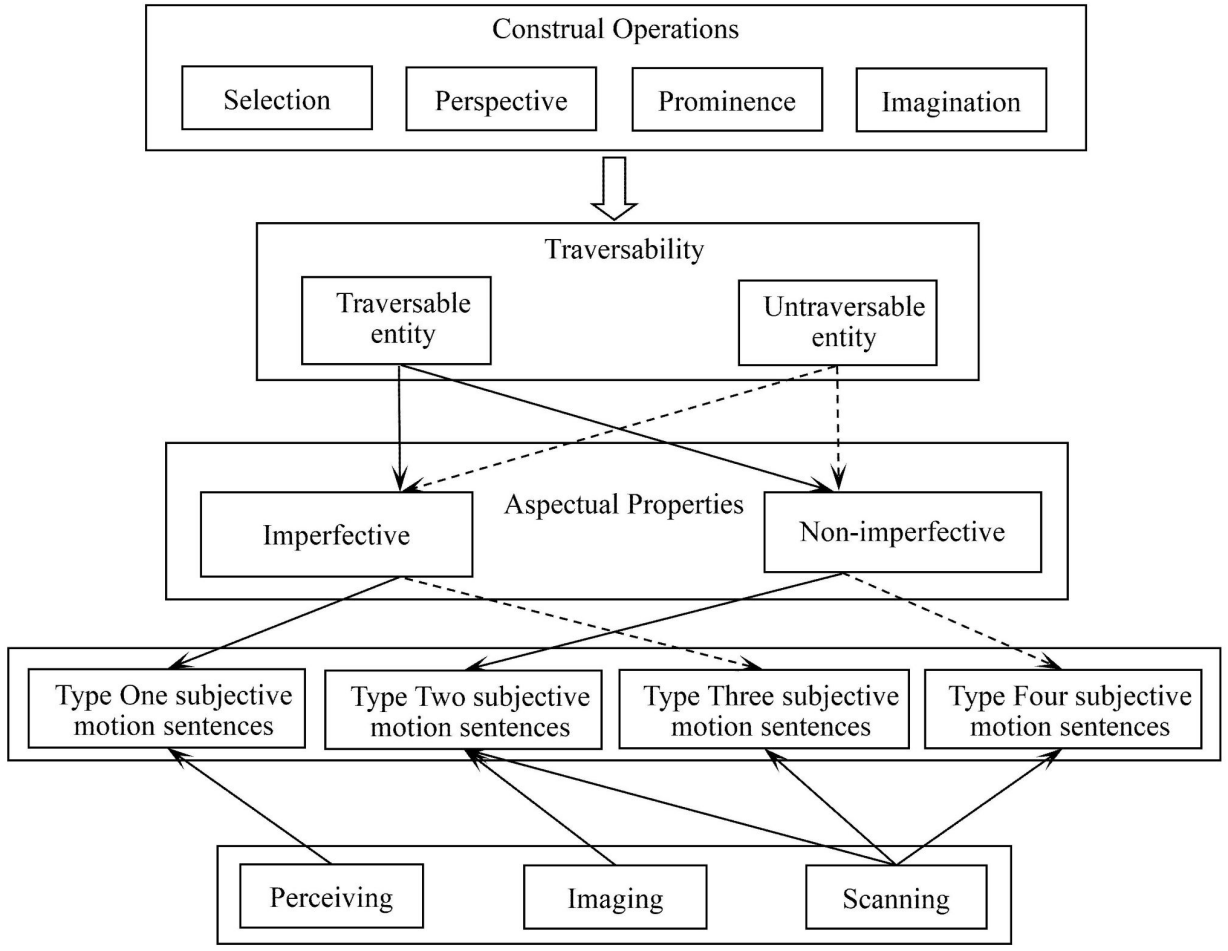
The framework for subjective motion production.

Based on the preceding analysis, we now propose a framework for subjective motion production. Notably, according to Langacker [21], the choice between distinct aspectual construals is not necessarily determined by the inherent properties of the scene described. In some cases, it depends on general or contextual knowledge; in others, it is simply a matter of speaker choice. That is to say, even if a static situation is observed from a first-person perspective, a speaker may still opt to produce a *Type Two subjective motion* sentence (i.e., non-imperfective). Furthermore, the same static situation can be construed through multiple sentences involving alternate choices regarding the level of precision, the direction of scanning, the selection of figure and ground, or the profiling of substructures. Therefore, these construal operations are posited as the guiding principles for the diverse lexicalizations of subjective motion. The proposed framework is illustrated in Fig 5.

Our pictorial stimuli are therefore intended to be in pairs, that is, depicting each entity type (traversable vs. untraversable) from both first-person and third-person perspectives. This design will operationalize the theoretical principle that aspectual construal varies according to the conceptualizer’s vantage point, with first-person perspective intended to induce an immersive, dynamic perspective (“within” the scene) and third-person perspective designed to facilitate a distanced perspective, as discussed earlier.

Interestingly, while developing a novel analytical framework grounded in cognitive construal, we discovered an unexpected alignment with Blomberg’s stimulus parameters – subject traversability (termed “Affordance” by Blomberg) and perspective [35]. However, the contribution of the present study extends beyond the selection of these dimensions per se. Instead, it lies in the construction of a construal-based analytical layer that: (1) explicitly predicts aspectual choices in Mandarin, (2) yields a comprehensive four-way typology integrating aspect and traversability, and (3) systematically links each type to hypothesized experiential motivations. This theoretical integration provides a mechanism for replicable cross-linguistic comparison, enabling a finer-grained analysis of motion construal than phenomenological approaches [17] alone.

### Methods

#### Stimuli.

Despite theoretical differences, the alignment in stimulus design led us to adopt Blomberg’s picture set [35] for cross-study comparability. However, existing applications of his stimuli (comprising 24 target pictures, 12 controls and 2 warm-up pictures) revealed critical limitations: “some pictures were more clearly proximal or distal than others, or some were more clearly motion-affording than others” [18]. Furthermore, the stimulus set exclusively featured linear, spatially extended entities, omitting other geometric configurations (e.g., curved or zigzag-shaped objects).

**Fig 6:**
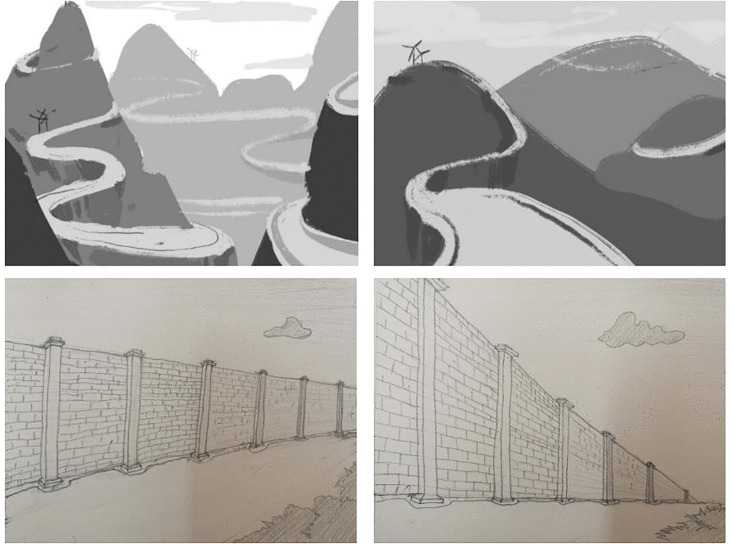
Sample stimuli.

To address these issues, we propose targeted modifications to the original design. Specifically, we removed 2 target pairs (four pictures) with ambiguous proximal/distal distinctions, and added 6 new target pairs (12 pictures) incorporating diverse shapes. Consequently, the final stimulus set used in this study consisted of 32 target pictures (16 pairs) and 12 control pictures, totaling 44 experimental items. Fig 6 presents sample stimuli from the modified set:

#### Participants.

A total of 100 native Mandarin speakers (47 males, 53 females) participated in the elicitations. All participants were undergraduate or graduate students at University of South China and received either course credit or movie tickets in return for their participation. All of the Chinese speakers have Mandarin as their first language; also, it is their only language until at least the age of eight, with an average age at the onset of English acquisition of twelve years. Participant recruitment took place from April 1^st^ to May 1^st^, 2022. All procedures involving human participants were conducted in accordance with the Declaration of Helsinki and were approved by the Academic Ethics Committee of the School of Languages and Literature, University of South China (No. USC20220330−001). Written informed consent was obtained from all participants prior to participation.

#### Procedure and statistical analysis.

During the experiment, participants were instructed to describe each picture’s spatial relations in one complete sentence, without starting with “I see...” or “The picture shows...”. All sessions were audio-recorded and transcribed. Two trained researchers independently coded the data in three steps: (1) screening for valid spatial descriptions (excluding training items and fragments); (2) classifying valid responses as subjective motion vs. non-subjective motion; and (3) assigning subjective-motion responses to one of the four proposed types. Discrepancies at any stage were resolved through discussion with reference to the coding manual until consensus was reached.

Statistical analyses were conducted using IBM SPSS Statistics (version 27.0). We report test statistics (χ², *df*, *p*) and effect sizes for all comparisons. Task validity was assessed by comparing subjective-motion rates between Target and Control stimuli using Fisher’s exact test (reporting OR). We then restricted the dataset to subjective motion responses to evaluate: (1) the frequency distribution of the four types using a Chi-square goodness-of-fit test (reporting Cohen’s *w*) and (2) the association between Aspect (imperfective vs. non-imperfective) and Traversability (traversable vs. untraversable) using a 2 × 2 Chi-square test of independence (reporting Cramer’s *V*). Finally, using responses to Target stimuli only, we tested whether Traversability is associated with subjective motion production (produced vs. not produced) via a separate 2 × 2 Chi-square test of independence (reporting Cramer’s *V*).

### Results

After excluding training items and invalid responses, we obtained a total of 3845 valid spatial descriptions: 2829 for target pictures and 1016 for control pictures. This substantial dataset indicates that participants complied well with the task instructions.

Descriptive statistics show that participants clearly differentiated target from control stimuli. As shown in Table 1, 36.23% of the descriptions for target stimuli (1025 out of 2829) contained subjective motion sentences. This contrasts sharply with the control stimuli, which elicited subjective motion sentences in less than 0.4% of cases (only 4 out of 1016). A Fisher’s exact test confirmed a highly significant association between stimulus type and sentence production (*p* < .001, OR = 143.75), demonstrating that the target stimuli successfully and reliably elicited subjective motion sentences compared to controls. Descriptively, the subjective motion production rate in Mandarin is in the same range as reported for Swedish, French and Thai using comparable picture-elicitation paradigms [35] (approximately 40%). While we treat this comparison as contextual rather than inferential, it suggests that Mandarin speakers readily recruit subjective motion descriptions in this elicitation setting.

**Table 1:** Subjective motion and non-subjective motion sentences for target and control stimuli.

Stimuli Type Response Category	Target Stimuli	Control Stimuli
Subjective Motion Sentences	1025	4
Non-subjective Motion Sentences	1804	1012
Total	2829	1016

Instances of all four proposed types were identified within the elicited subjective motion sentences (Table 2), demonstrating that the framework-guided design successfully elicited the full theoretical range of subjective motion expressions. To assess how these types are distributed, we conducted a Chi-square goodness-of-fit test. The results indicate that the four types are not equally represented (χ² = 1047.0, *df* = 3, *p* < .001, Cohen’s *w* = 1.01). This non-uniformity is largely attributable to a strong dominance of non-imperfective realizations (Types Two and Type Four), which together account for 94.92% of the observed subjective motion sentences.

**Table 2:** Distribution of subjective motion sentences per sentence type in elicitation experiment.

Sentence Type	Type One	Type Two	Type Three	Type Four	Total
Number	34	654	18	319	1025
Percentage	3.32%	63.80%	1.76%	31.12%	N/A

To evaluate whether aspectual marking varies by entity type, we then performed a 2 × 2 contingency analysis on the subjective-motion subset (Traversability × Aspect; imperfective vs. non-imperfective). The analysis showed no significant association (χ² = 0.015, *df* = 1, *p* = .903, Cramer’s *V* = .004): imperfective marking was comparably rare for both traversable (4.9%) and untraversable (5.3%) entities. In other words, within Mandarin subjective-motion productions, aspect choice appears statistically independent of traversability in this dataset. This pattern is compatible with the characterization of subjective motion as typically describing stable, atemporal configurations [30]: even when the figure is traversable (e.g., a road), speakers most often construe the scene configurationally rather than as an unfolding process, and therefore seldom recruit overt imperfective markers.

Finally, to test whether traversability is associated with the likelihood of producing subjective motion (as opposed to aspect choice within subjective motion), we conducted a separate 2 × 2 contingency analysis (Traversability × Production). As shown in Table 3, the association was significant (χ² = 192.7, *df* = 1, *p* < .001, Cramer’s *V* = .26). Specifically, traversable entities elicited subjective motion sentences at a higher rate (48.83%) compared to untraversable entities (23.73%). This result converges with previous cross-linguistic reports [2,4,11,35] and supports the interpretation that traversability is a key factor associated with subjective-motion production in this elicitation task.

**Table 3:** Production of subjective motion sentences by entity traversability.

Entity Type	Subjective Motion Sentences	Non-Subjective Motion Sentences	Total	Production Rate
Traversable	688	721	1409	48.83%
Untraversable	337	1083	1420	23.73%

It is important to note that the primary objective of this study was to elicit a substantial dataset of subjective motion sentences to facilitate cross-linguistic comparison. Consequently, the current analysis focuses exclusively on reporting the overall production rate and the frequency distribution of distinct sentence types in Mandarin. A more granular investigation into the lexical diversity and frequency of path and manner information within each type, as well as the underlying experiential motivations governing these linguistic choices, will be systematically examined in future research.

## Conclusion

Many questions about subjective motion remain unresolved, particularly concerning how it is linguistically represented in natural discourse, when people mentally scan along a figure versus move along it, and how subjective motion interacts with diverse aspectual patterns. These questions are related to challenges facing the production of subjective motion sentences. To address these challenges, this study proposes an experimental framework that advances production research by operationalizing cognitive construal for subjective motion conceptualization, establishing a systematic classification of sentence types based on aspectual marking and subject traversability, and identifying their underlying cognitive motivations.

However, it is important to acknowledge a limitation regarding the empirical verification of these motivations. While our stimulus design was grounded in theoretical postulates regarding spatial access, the current study relied on offline linguistic production data rather than online processing measures. We did not employ techniques such as eye-tracking or reaction times to confirm that speakers were indeed engaging in the specific cognitive operations (e.g., sequential scanning) hypothesized for each condition. Therefore, the connection between the visual input and the linguistic output remains inferential. Future research combining corpus analysis with eye-tracking experiments would be instrumental in empirically validating these real-time cognitive mechanisms.

Using Mandarin as our case study, we present this approach not as a universal model, but as a preliminary framework with potential for cross-linguistic adaptation. Future testing of this framework in tense-prominent languages or other typologically distinct languages (e.g., Spanish, Hindi) is essential to determine its wider applicability. By extending this line of inquiry, we hope to further elucidate language-specific patterns and the universal cognitive mechanisms underlying why, how, and when speakers produce subjective motion sentences.
